# Efficacy and safety of preoperative 5-fluorouracil, cisplatin, and mitomycin C in combination with radiotherapy in patients with resectable and borderline resectable pancreatic cancer: a long-term follow-up study

**DOI:** 10.1186/s12957-019-1687-4

**Published:** 2019-08-16

**Authors:** Yutaka Endo, Minoru Kitago, Koichi Aiura, Masahiro Shinoda, Hiroshi Yagi, Yuta Abe, Go Oshima, Shutaro Hori, Yutaka Nakano, Osamu Itano, Junichi Fukada, Yohei Masugi, Yuko Kitagawa

**Affiliations:** 1 Department of Surgery, Keio University School of Medicine, 35 Shinanomachi, Shinjuku-Ku, Tokyo, 160-8582 Japan; 2 Department of Surgery, Kawasaki City Hospital, Kanagawa, Japan; 3 Department of Gastrointestinal Surgery, International University of Health and Welfare, Chiba, Japan; 4 Department of Radiology, Keio University School of Medicine, Tokyo, Japan; 5 Department of Pathology, Keio University School of Medicine, Tokyo, Japan

**Keywords:** Chemoradiotherapy, Follow-up studies, Neoadjuvant therapy, Pancreatic carcinoma

## Abstract

**Background:**

We aimed to evaluate the efficacy and safety of 5-fluorouracil-based neoadjuvant chemoradiotherapy (NACRT) in patients with resectable/borderline resectable pancreatic ductal adenocarcinoma (PDAC).

**Methods:**

This retrospective study investigated the clinicopathological features and > 5-year survival of patients with T3/T4 PDAC who underwent NACRT at our institute between 2003 and 2012.

**Results:**

Seventeen resectable and eight borderline resectable patients were included. The protocol treatment completion and resection rates were 92.0% and 68.0%, respectively. Two patients failed to complete chemotherapy owing to cholangitis or anorexia. Common grade 3 toxicities included anorexia (12%), neutropenia (4%), thrombocytopenia (4%), anemia (4%), and leukopenia (12%). Pathologically negative margins were achieved in 94.1% of patients who underwent pancreatectomy. Pathological response according to Evans’ classification was grade IIA in 10 patients (58.8%), IIB in 5 patients (29.4%), and IV in 2 patients (11.8%). Postoperative pancreatic fistulas were observed in four patients (23.5%), delayed gastric emptying in one patient (5.9%), and other operative morbidities in four patients (23.5%). The 1-, 2-, 5-, and 10-year overall survival rates were 73.9%, 60.9%, 60.9%, and 39.1%, respectively (median follow-up period, 80.3 months).

**Conclusions:**

NACRT is tolerable and beneficial for resectable/borderline resectable PDAC, even in the long-term.

## Introduction

Pancreatic cancer, especially pancreatic ductal adenocarcinoma (PDAC), is a devastating disease that is associated with poor prognosis and low resectability rates (15.0–20.0%) [1]. When possible, surgical resection is the only curative treatment available. However, approximately 80.0% of patients experience recurrence after a short time interval, with a median survival of approximately 20 months [2]. Because of the minimal survival benefit of surgery alone, adjuvant and neoadjuvant treatment strategies for PDAC are being actively investigated. There have been several reports regarding the efficacy of adjuvant therapies for resected pancreatic cancer [3, 4]. However, the ideal neoadjuvant treatment protocol and its significance for prognosis remain unclear [5].

One rationale for using neoadjuvant chemotherapy or neoadjuvant chemoradiotherapy (NACRT) is to achieve negative resection margins (R0) because survival rates are poor in patients with positive resection margins (R1/R2). Another reason is its more effective delivery, compared to adjuvant chemotherapy, without potential delays caused by surgical complications. The proposed benefits of chemoradiotherapy in pancreatic cancer are local disease control and improved rates of complete resection [6–8]. Katz et al. [9] reported that preoperative chemoradiotherapy was associated with a median survival of 40 months in resected patients. However, the overall survival (OS) benefits of NACRT remain unclear.

We have administered NACRT using 5-fluorouracil (5-FU), cisplatin, and mitomycin C in combination with radiotherapy since the early 2000s. The rationale for our regimen was that there were several reports concerning to the anti-tumor effect of mitomycin C and cisplatin in the combination of 5-FU [10, 11]. However, there have been no reports concerning the long-term effects of NACRT for PDAC. Therefore, we aimed to evaluate the short-term safety and long-term efficacy of NACRT for potentially resectable PDAC in a long-term follow-up study.

## Methods

Twenty-five patients who underwent NACRT and subsequent surgery at Keio University Hospital (Tokyo, Japan) between May 2003 and August 2012 were retrospectively analyzed to evaluate the efficacy and safety of NACRT. NACRT was selectively administered to a limited number of patients with T3/T4 PDAC according to the Tumor-Node-Metastasis classification, seventh edition, who agreed with this treatment. In addition, selected patients had a performance status of 0–1, were 20–80 years of age, and had adequate organ function (defined by no abnormal laboratory findings for chemotherapy). Prior to NACRT and surgery, all patients underwent staging investigations to examine evidence of distant metastasis by contrast-enhanced computed tomography (CT) or magnetic resonance imaging. Preoperative cytologic confirmation was not mandatory if the patients’ lesions were highly suspected to be pancreatic cancer. PET scan and laparoscopy were not used for staging. We conducted a retrospective observational study and used the “opt-out” method as a way to obtain informed consent from patients. The study was approved by the Human Experimentation Committee of our institution (no. 20120279).

The NACRT regimen consisted of a combination of 4 cycles of chemotherapy (continuous administration of 5-FU; cisplatin on day 5, 12, 19, and 26; mitomycin C on day 6, 13, 20, and 27; and heparin infusion) and radiotherapy (planned total dose, 40.0 Gy of external beam radiation therapy [40.0 Gy per 20 fractions]). After completing NACRT, patients underwent restaging CT to determine resectability. Approximately 1–2 weeks after completing NACRT, patients without evidence of disease progression and who were medically fit were taken into the operating room for subsequent curative surgery. All adverse events experienced during the study were recorded and graded according to the National Cancer Institute Common Terminology Criteria for Adverse Events (version 4.0). Radiological responses in patients who underwent NACRT were evaluated by CT using the Response Evaluation Criteria in Solid Tumors [12].

Surgery, which included pylorus-preserving or subtotal stomach-preserving pancreatoduodenectomy or distal pancreatectomy accompanied by extensive lymphatic and connective tissue clearance in combination with or without postoperative liver perfusion chemotherapy and adjuvant chemotherapy, was performed as described previously [13]. The postoperative morbidity rate included all complications following surgery (classified according to the Clavien-Dindo classification [14]) up to the day of discharge. A postoperative pancreatic fistula (POPF) was defined according to the criteria of the International Study Group on Pancreatic Fistula [15], and delayed gastric emptying was defined according to the criteria of the International Study Group of Pancreatic Surgery [16]. A POPF of grade B/C was considered a clinically significant complication.

Pathological responses in patients who underwent NACRT were evaluated based on the proportion of residual viable tumor cells according to the classification proposed by Evans et al. [17]. Pathological data obtained also included the Tumor-Node-Metastasis classification, the surgical margin status, the presence or absence of microscopic lymphovascular and perineural invasion, the tumor differentiation, and the presence or absence of major vascular invasion. The surgical margin represented either the pancreatic or bile duct stump or the dissected plane around the pancreas. If viable microscopic cancer cells were detected at the edge of these sites, the surgical margin status was considered positive [18, 19].

After surgical resection of the PDAC, each patient received the standard postoperative follow-up. Recurrence was defined by definitive evidence of recurrence, which was confirmed with radiographic findings, with or without elevated serum cancer antigen 19-9 levels. Physical examinations, toxicity assessments, complete blood cell counts, serum chemistry profiles, and chest-abdominal CT scans were performed approximately every 4–6 months for the first 12 months and every 6 months thereafter.

### Statistical analyses

Survival curves were plotted using the Kaplan-Meier method and compared using the log-rank test. OS was defined as the time interval between the date of commencing preoperative therapy and the date of death from any cause or last follow-up. For patients who underwent surgical resection, recurrence-free survival was defined as the time interval between the date of surgery and the date of first recurrence (local, distant, or both) or death, whichever occurred first. All statistical analyses were conducted using JMP 12 (SAS Institute Inc., Cary, NC, USA).

## Results

### Clinical characteristics

Table 1 summarizes the patients’ clinical characteristics before the commencement of NACRT. Twenty-five patients with potentially resectable (*n* = 17) or borderline resectable (*n* = 8) pancreatic cancer were investigated. The eight borderline resectable patients included six patients with portal vein invasion and two patients with arterial abutment.

**Table 1:** Patients’ characteristics

Characteristic	Patients (*n* = 25)
Age (years), median (range)	66 (51–80)
Tumor size (mm), median (range)	28 (12–40)
Sex, *n* (%)	
Male	16 (64.0)
Female	9 (36.0)
Primary tumor location, *n* (%)	
Head/neck	18 (72.0)
Body	6 (24.0)
Tail	1 (4.0)
NCCN resectability, *n* (%)	
Resectable	17 (68.0)
Borderline resectable	8 (32.0)
BR-PV	6 (24.0)
BR-A	2 (8.0)
Completion of NACRT, *n* (%)	23 (92.0)
Completion of RT, *n* (%)	25 (100.0)
Completion of CT, *n* (%)	23 (92.0)
Resection rate, *n* (%)	17 (68.0)
Reason for protocol failure, *n* (%)	
Cholangitis	1 (4.0)
Neutropenia	1 (4.0)

*Abbreviations*: *CT* chemotherapy, *NACRT* neoadjuvant chemoradiotherapy, *NCCN* National Comprehensive Cancer Network, *BR-PV* borderline resectable-portal vein, *BR-A* borderline resectable-artery, *RT* radiotherapy

### Treatment responses

**Fig. 1:**
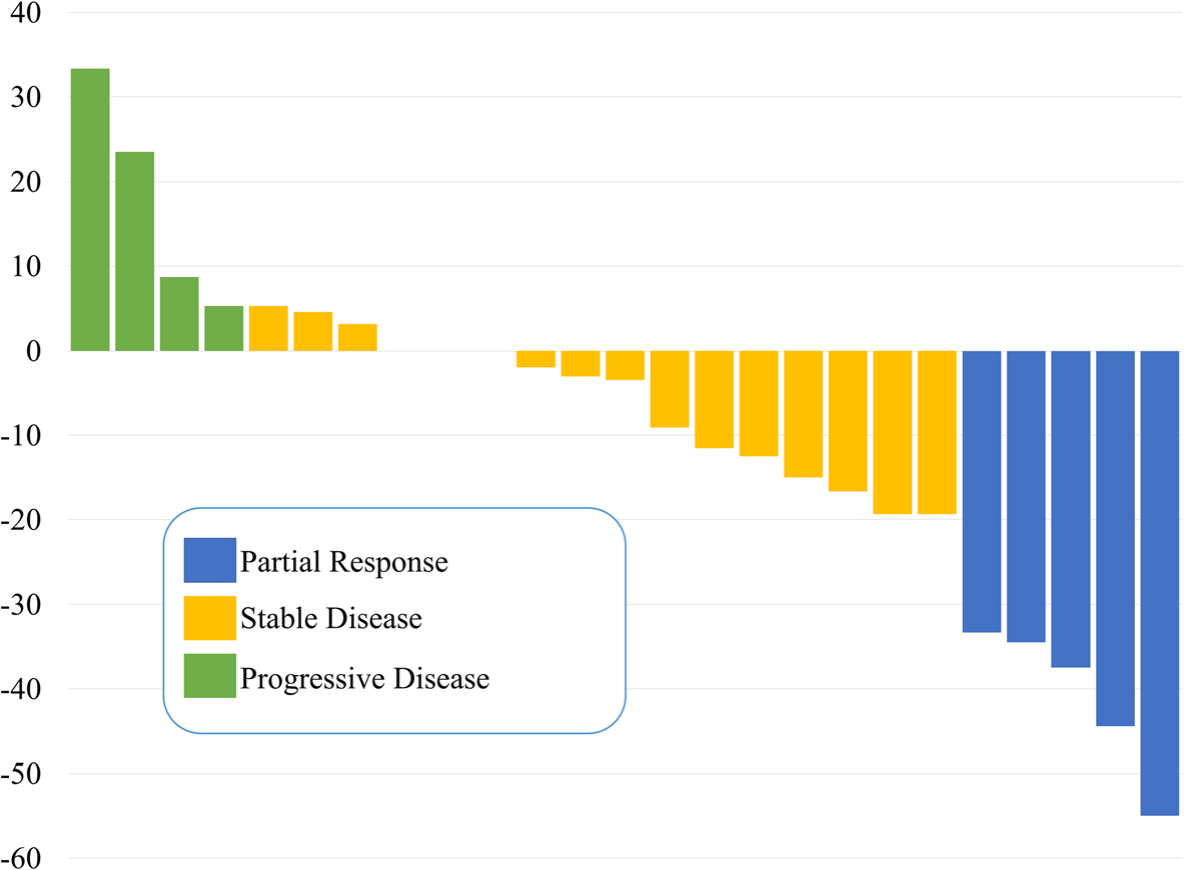
Waterfall plot of maximum percentage change from baseline during neoadjuvant chemoradiotherapy

The radiological responses to NACRT are shown in Fig. 1. The waterfall plot of the maximum percentage change of the primary site from baseline during NACRT identified 16 patients (16/25, 64.0%) with stable disease, 5 patients (5/25, 20.0%) with partial response, and 4 patients (4/25, 16.0%) with progressive disease. Four patients with progressive disease, who developed liver metastases that were detected during preoperative assessment with multidetector CT and surgery, did not undergo resection. Two patients with macroscopic peritoneal dissemination during surgery did not undergo resection. One patient with local disease progression underwent gastrojejunal bypass surgery. One patient with reduced performance status did not undergo resection.

Of the 17 patients (17/25, 68.0%) who underwent tumor resection, 13 (13/17, 76.4%) patients underwent pancreatoduodenectomy and 4 (4/17, 23.5%) patients underwent distal pancreatectomy. None of the patients underwent total pancreatectomy. Portal vascular resection was performed in four patients (4/17, 23.5%). None of the patients underwent hepatic or celiac artery resection. The median operative time for pancreatoduodenectomy was 678 (range, 372–1032) min, with a median estimated blood loss of 785.0 (range, 120.0–2390.0) mL. The median operative time for distal pancreatectomy was 437 (range, 387–648) min, with a median estimated blood loss of 217.5 (range, 100.0–1210.0) mL.

### Toxicity and complications during NACRT and subsequent surgery

NACRT-related toxicities are summarized in Table 2. During NACRT, there was no NACRT-related mortality. Grade 3 neutropenia, leukopenia, anemia, thrombocytopenia, and anorexia occurred in zero, three, one, zero, and two patients, respectively. The protocol treatment completion and resection rates were 92.0% (23/25) and 68.0% (17/25), respectively (Table 1). Two patients failed to complete chemotherapy owing to cholangitis (1/25, 4.0%) or anorexia (1/25, 4.0%). All patients received the planned dose of radiotherapy. Among the 17 patients who underwent resection, clinically significant POPFs were observed in 4 patients (4/17, 23.5%), delayed gastric emptying was observed in 1 patient (1/17, 5.9%), and other operative morbidities (Clavien-Dindo grade IIIA or higher) were observed in 4 patients (4/17, 23.5%). None of the patients required further surgery. Furthermore, 8 of the patients who underwent resection (8/17, 47.0%) received portal vein infusion for 4 weeks immediately after surgery, 2 (2/17, 11.8%) patients received adjuvant chemotherapy (5-FU, etc.), and 5 (5/17, 29.4%) patients received both.

**Table 2:** Toxicity profiles

Toxicity	Grade (CTCAE v4.0)					
	1	2	3	4	All	G3
Hematological						
Leukopenia	1	12	3	0	16	3
Neutropenia	0	2	0	0	2	0
Anemia	4	2	1	0	7	1
Thrombocytopenia	0	1	0	0	1	0
Non-hematological						
Elevated creatinine	0	0	0	0	0	0
Elevated AST/ALT	2	0	0	0	2	0
Hyperbilirubinemia	0	0	0	0	0	0
Hyponatremia	2	0	0	0	2	0
Alopecia	0	0	0	0	0	0
Anorexia	3	2	2	0	7	2
Constipation	0	0	0	0	0	0
Diarrhea	0	0	0	0	0	0
Edema	0	0	0	0	0	0
Fever	0	0	0	0	0	0
Nausea	0	0	0	0	0	0
Rash	0	0	0	0	0	0
Stomatitis	0	0	0	0	0	0
Vomiting	0	0	0	0	0	0

*Abbreviations*: *AST* aspartate aminotransferase, *ALT* alanine aminotransferase, *CTCAE* Common Terminology Criteria for Adverse Events, *G* grade, *v* version

### Pathological findings of NACRT

The pathological findings in the 17 patients who underwent resection are summarized in Table 3. Pathological evaluation revealed that all patients had PDAC. Five patients had node-positive disease, and two patients had portal vein invasion. None of the patients had major arterial invasion. Pathological response according to Evans’ classification was grade IIA in 10 patients (10/17, 58.8%), IIB in 5 patients (5/17, 29.4%), and IV in 2 patients (2/17, 11.8%).

**Table 3:** Pathological characteristics

Characteristic	Patients (*n* = 17)
Histology (PDAC), *n* (%)	17 (100.0)
T stage, *n* (%)	
T0	0 (0.0)
Tis	0 (0.0)
T1	4 (23.5)
T2	1 (5.9)
T3	12 (70.6)
N stage, *n* (%)	
N0	12 (70.6)
N1	5 (29.4)
TNM stage, *n* (%)	
0	0 (0.0)
IA	3 (17.6)
IB	1 (5.9)
IIA	8 (47.1)
IIB	5 (29.4)
Negative microscopic resection margins, *n* (%)	
R0	16 (94.1)
R1	1 (5.9)
Differentiation, *n* (%)	
Well-moderate	6 (35.3)
Moderate-poor	10 (58.8)
Other	1 (5.9)
Portal vein invasion status, *n* (%)	2 (11.8)
Microscopic lymphovascular invasion, *n* (%)	8 (47.1)
Microscopic perineural invasion, *n* (%)	5 (29.4)
Evans’ classification, *n* (%)	
I	0 (0.0)
IIA	10 (58.8)
IIB	5 (29.4)
III	0 (0.0)
IV	2 (11.8)

*Abbreviations*: *PDAC* pancreatic ductal adenocarcinoma, *TNM* tumor-node-metastasis

### Survival analyses

**Fig. 2:**
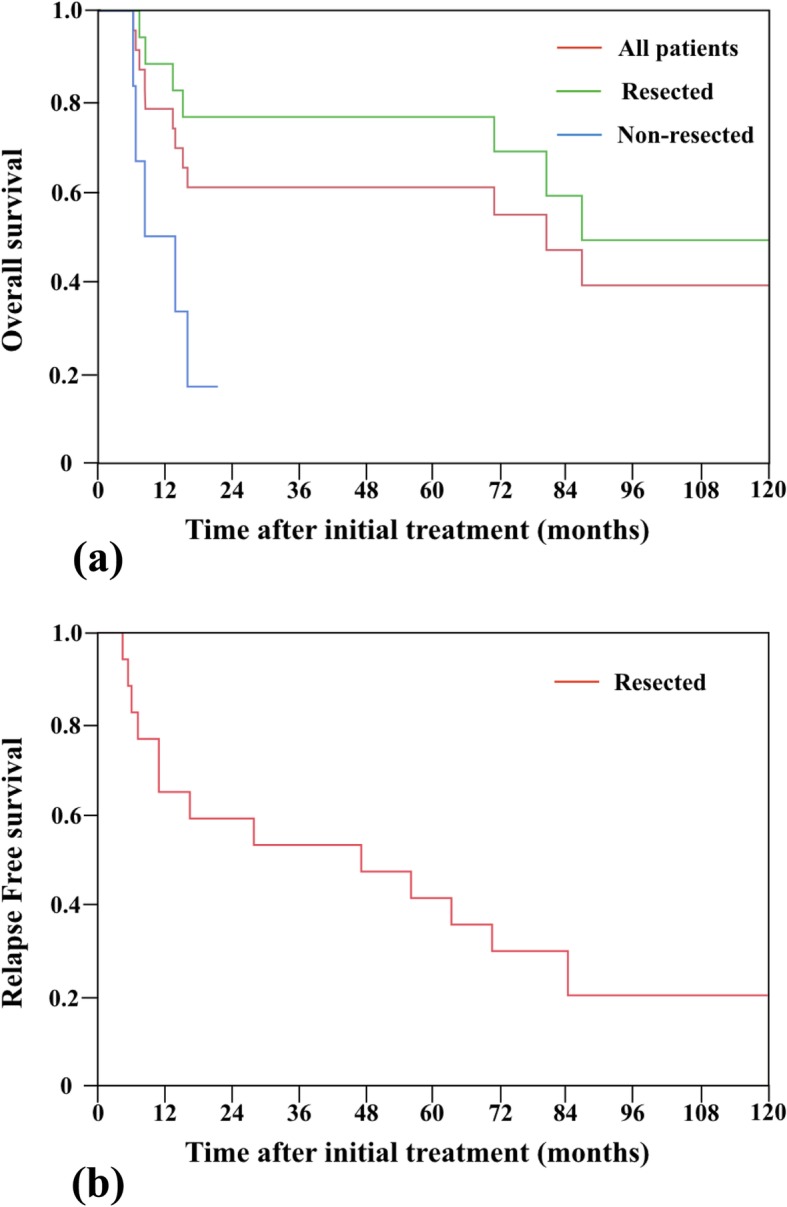
Kaplan-Meier curves of **a** overall survival in patients receiving neoadjuvant chemoradiotherapy and **b** recurrence-free survival in patients who underwent surgical resection

The 1-, 2-, 5-, and 10-year OS rates for all patients combined were 73.9%, 60.9%, 60.9%, and 39.1%, respectively, with a median follow-up period of 80.3 (range, 2.6–145.0) months. The 1-, 2-, 5-, and 10-year survival rates for the resected cases were 82.3%, 76.5%, 76.5%, and 49.2%, respectively, for OS and 64.7%, 58.8%, 52.9%, and 19.6%, respectively, for recurrence-free survival (Fig. 2a–b). Recurrence was noted in 10 (52.9%) of the 17 patients who underwent resection. Patterns of recurrence included distant metastasis in seven patients (70.0%), local recurrence in two patients (20.0%), and remnant pancreatic cancer in one patient (10.0%). Ten patients (10/25, 40.0%) survived for ≥ 5 years; four patients (4/25, 16.0%) survived for > 5 years without any signs of recurrence.

## Discussion

This study is the first to evaluate the short-term safety and long-term efficacy of NACRT using 5-FU, cisplatin, and mitomycin C in combination with radiotherapy for 5 years or more. We observed a relatively high survival rate after subsequent surgery with low toxicity. The overall toxicity profile of this regimen was fully acceptable without any grade 4 toxicities. However, the incidence of postoperative complications, especially POPF grade B/C (4/17, 23.5%), was relatively high compared to that of previous reports [20, 21], which demonstrated an 11–17% rate of POPF. There is one potential explanation for this finding. Compared to the early 2000s when the operation in this analysis was performed, there has been notable progress in the pancreatic anastomosis procedure and in both intra- and postoperative management [22]. These recent advances may account for the discrepancy between the POPF rate in our study and those of our recent surgical results.

Since we had followed the patients analyzed in this study for > 5 years, we were able to calculate actual 5-year survival rates. In the present study, 10 patients (10/25, 40.0%) survived for ≥ 5 years and 4 patients (4/25, 16.0%) survived for > 5 years without any signs of recurrence. Compared to previous studies [23–25], the actual 5-year survival rates in our study seemed to be favorable. Moreover, there were no late adverse effects of NACRT (e.g., secondary tumorigenesis, endocrinological disturbance, or retroperitoneal fibrosis). Studies evaluating long-term follow-up after pancreatectomy and NACRT are scarce. In the present study, we have shown that NACRT is a potent and safe strategy for treating patients with PDAC, even in the long-term. There may be several reasons for this. First, the present study [26] included two patients who experienced complete remission and achieved 5-year survival. There have been several reports [27, 28] of pathological complete remission with neoadjuvant therapy, such as 5-FU- and gemcitabine-based regimens with or without radiotherapy, in patients with PDAC, with rates of 3.3% and 7.0%, respectively. Pathological tumor response in post-therapy specimens may be used as a successful surrogate for longer recurrence-free survival in patients with resectable PDAC. Mellon et al. [29] recently demonstrated that patients with pathological complete response had superior outcomes. Secondly, we adopted postoperative portal vein infusion chemotherapy as described previously [13]. Therefore, owing to a combination of NACRT and portal vein infusion chemotherapy, we could control the major causes of treatment failure (i.e., local recurrence and liver metastasis). This hypothesis is supported by our previous report [30] concerning clinical variables associated with > 5-year survival after pancreatectomy and identifying both NACRT and portal vein infusion chemotherapy as positive prognostic factors. Also, both NACRT and portal vein infusion regimens included heparin, which is suspected to have anti-tumor activity, according to the previous studies [31]. Therefore, heparin might add an anti-malignant effect. Lastly, the number of patients with positive lymph node metastasis was relatively low (5/17, 29.4%), so this contributed to the better outcome of this study. A recent systematic review of the association between neoadjuvant therapy and its pathological characteristics demonstrated a beneficial effect of lower rate of lymph node metastasis [32].

Previous studies concerning 5-FU-based NACRT have been published [17, 33–36]. According to these studies, the resection rate is approximately 60.0–80.0% and the proportion of patients who achieve complete response is approximately 0.0–8.0%. Therefore, data on the resection rate and histopathological assessment of NACRT effects with 5-FU, cisplatin, and mitomycin C suggest that our strategy is as effective as those previously reported [17, 33–36]. However, the resection rate was relatively low compared to recently published reports of patients receiving NACRT [21, 37]. This could primarily be because the detection ability of the CT scan at the time of the present study was inaccurate, meaning that small metastatic lesions could not be detected on initial workup. Positron emission tomography-CT or gadoxetic acid-enhanced magnetic resonance imaging is now available in the clinical setting, enabling clinicians to distinguish more precisely between patients with and without metastatic disease. However, considering this transition in radiographic modality, there is still room for improvement in our NACRT regimen. Recent studies [38, 39] have demonstrated that more active combinations, such as FOLFIRINOX (leucovorin, 5-FU, irinotecan, and oxaliplatin) or gemcitabine and nab-paclitaxel, have strong anti-tumor effects. Therefore, these may be candidates for improving preoperative therapy and resection rates.

This study has several limitations. First, the study was retrospective in nature and had a single-center design; therefore, the results lacked external validity. Second, the number of enrolled patients was limited. Third, there is a possibility that our analyzed patients had indolent diseases, and therefore, our relatively favorable survival rate might be affected by selection bias. Therefore, this study was not designed to prove the survival benefit of NACRT. Further, multicenter studies with proper patient selection and larger sample sizes are warranted to achieve a robust conclusion.

In conclusion, preoperative administration of 5-FU, cisplatin, and mitomycin C in combination with radiotherapy is well tolerated and safe. This is the first study to evaluate the efficacy and safety of NACRT using 5-FU, cisplatin, mitomycin C, and heparin in combination with radiotherapy in the long-term. Our protocol achieved a relatively high survival rate after subsequent surgery.

## Data Availability

The datasets used and analyzed during the current study are available from the corresponding author on reasonable request.

## Abbreviations

NACRT: Neoadjuvant chemoradiotherapy
PDAC: Pancreatic ductal adenocarcinoma
POPF: Postoperative pancreatic fistula
